# Carbon-ion radiotherapy for cholangiocarcinoma: a multi-institutional study by and the Japan carbon-ion radiation oncology study group (J-CROS)

**DOI:** 10.18632/oncotarget.27028

**Published:** 2019-07-09

**Authors:** Goro Kasuya, Kazuki Terashima, Kei Shibuya, Shingo Toyama, Daniel K. Ebner, Hiroshi Tsuji, Tomoaki Okimoto, Tatsuya Ohno, Yoshiyuki Shioyama, Takashi Nakano, Tadashi Kamada, the Japan Carbon-Ion Radiation Oncology Study Group

**Affiliations:** ^1^ QST Hospital (Former Hospital of the National Institute of Radiological Sciences), National Institutes for Quantum and Radiological Science and Technology, Chiba, Japan; ^2^ Department of Radiology, Hyogo Ion Beam Medical Center, Tatsuno, Japan; ^3^ Gunma University Heavy Ion Medical Center, Maebashi, Japan; ^4^ Ion Beam Therapy Center, SAGA-HIMAT Foundation, Tosu, Japan; ^5^ Harvard TH Chan School of Public Health, Boston, MA, USA; ^6^ Department of Radiology, Kyushu University, Hakata, Japan

**Keywords:** carbon-ion radiotherapy, radiation, cholangiocarcinoma, adverse event, survival

## Abstract

To evaluate the safety and efficacy of carbon-ion radiotherapy (CIRT) for cholangiocarcinoma via a multicenter retrospective study. Clinical data were collected from patients with cholangiocarcinoma who had received CIRT at one of four treating institutions in Japan. Of 56 eligible patients, none received surgery for cholangiocarcinoma before or after CIRT. The primary endpoint was overall survival (OS). Based on the tumor site, the 56 cases were categorized as intrahepatic cholangiocarcinoma (IHC) (n=27) or perihilar cholangiocarcinoma (PHC) (n=29). In all patients, the median tumor size was 37 (range, 15‒110) mm, and the most commonly prescribed dose was 76 Gy (relative biological effectiveness) in 20 fractions. The median survival was 14.8 (range, 2.1-129.2) months, and the 1- and 2-year OS rates were 69.7% and 40.9%, respectively. The median survival times of the patients with IHC and those with PHC were 23.8 and 12.6 months, respectively. Both univariate and multivariate analyses revealed that cholangitis pre-CIRT and Child‒Pugh class B were significant prognostic factors for an unfavorable OS. Of four patients who died of liver failure, one with IHC was suspected to have radiation-induced liver disease because of newly developed ascites, and died at 4.3 months post-CIRT. Grade 3 CIRT-related bile duct stenosis was observed in one IHC case. No other CIRT-related severe adverse events, including gastrointestinal events, were observed. These results suggest that CIRT yields relatively favorable treatment outcomes, especially for patients with IHC, and acceptable toxicities were observed in patients with cholangiocarcinoma who did not receive surgery.

## INTRODUCTION

Cholangiocarcinoma is relatively uncommon, accounting for only 15% of hepatic malignancies [1]. Standard radical therapy involves surgery, although few patients are candidates for curative surgical resection at the time of presentation [2]. Although the typical standard treatment for inoperable cholangiocarcinoma is chemotherapy, the median survival time (MST) even after combined chemotherapy, including gemcitabine and cisplatin, is reported to be at most approximately 1 year [3, 4]. Conventionally fractionated radiotherapy has been suggested as a combination tactic for unresectable cholangiocarcinoma, with and without chemotherapy, for potential prolongation of survival; however, the prognoses remain poor according to several retrospective studies [5, 6]. Recently, stereotactic radiotherapy (SBRT) has enabled delivery of higher-dose irradiation to the tumor than conventional X-ray radiotherapy, and the effectiveness of these novel techniques is promising [7, 8]. However, radiotherapy-related severe adverse events, particularly duodenal or gastric ulcers, are reported to occur 10‒20% of the time [9].

Carbon-ion radiotherapy (CIRT) offers a higher linear energy transfer and subsequently greater relative biological effectiveness (RBE) compared with photons. Moreover, the Bragg-peak and limited lateral scattering of the beam offer a superior dose delivery in comparison with photon irradiation, allowing increased dose delivery to the tumor while reducing the dose to healthy tissue [10–12]. However, there is only one study on CIRT for cholangiocarcinoma, which was based on a small cohort from a single institution [13]. As such, the efficacy and safety of CIRT for cholangiocarcinoma are poorly understood.

We conducted a retrospective multicenter study to evaluate the clinical outcomes of CIRT for cholangiocarcinoma, referred to as Japan Carbon-Ion Radiation Oncology Study Group (J-CROS 1703). The purpose of this study was to evaluate the efficacy and safety of CIRT for the treatment of cholangiocarcinoma.

## RESULTS

The patient, tumor, and treatment characteristics of all 56 eligible patients with cholangiocarcinoma comprising 27 patients with intrahepatic cholangiocarcinoma (IHC) and 29 patients with perihilar cholangiocarcinoma (PHC), are shown in Table 1. All patients were restaged according to the 7^th^ edition of the Tumor‒Node‒Metastasis staging system (International Union Against Cancer, 2009). The median tumor size was 37 (range, 15‒110) mm. Biopsy was performed in 35 patients (63%), all of whom were diagnosed with adenocarcinoma except 2 patients who were diagnosed with mixed hepatocellular carcinoma/cholangiocarcinoma. The remaining 21 patients were diagnosed by imaging (dynamic contrast enhanced computed tomography and/or magnetic resonance imaging) as well as detection of elevated tumor markers (CA19-9 and/or CEA). The most commonly prescribed CIRT dose was 76 Gy (RBE) in 20 fractions (biological effective dose [BED] of 105 when α/β = 10 was applied). Thirteen patients (23%) were treated previously with chemotherapy, one patient with concurrent chemotherapy, and one patient with radiofrequency ablation. However, all tumors that were treated with other therapies had progressed prior to CIRT. No patient received surgical treatment for cholangiocarcinoma in this study.

**Table 1:** Patient, tumor, and treatment characteristics

Factor		Total [*n* = 56] (%)	Intrahepatic cholangiocarcinoma [*n* = 27] (%)	Perihilar cholangiocarcinoma [*n* = 29] (%)	*p*
Age, years	Median, [range]	74 [43–87]	75 [57–87]	71 [43–86]	0.225^§^
Sex	Male	38 (68)	19 (70)	19 (66)	0.698^*^
	Female	18 (32)	8 (30)	10 (34)	
Operability	Operable	10 (18)	5 (19)	5 (17)	0.587^¶^
	Inoperable	46 (82)	22 (81)	24 (83)	
Performance status	0/1	52 (93)	26 (96)	26 (90)	0.333^¶^
	2	4 (7)	1 (4)	3 (10)	
Child‒Pugh class	A	52 (93)	26 (96)	26 (90)	0.333^¶^
	B	4 (7)	1 (4)	3 (10)	
Prior therapy for cholangiocarcinoma	Yes	13 (23)	6 (22)	7 (24)	0.865^*^
	No	43 (77)	21 (78)	22 (76)	
Cholangitis pre-CIRT	Yes	11 (20)	0 (0)	11 (38)	> 0.001^¶^
	No	45 (80)	27 (100)	18 (62)	
Biliary stenosis pre-CIRT	Yes	27 (48)	0 (0)	27 (93)	>0.001^¶^
	No	29 (52)	27 (100)	2 (7)	
Stent treatment for biliary stenosis pre-CIRT	Yes	24 (43)	0 (0)	24 (83)	>0.001^¶^
	No	32 (57)	27 (100)	5 (17)	
Diagnosis	Pathological	35 (63)	17 (63)	18 (62)	0.945^*^
	Imaging + tumor markers	21 (37)	10 (37)	11 (38)	
TNM classification	T1N0M0	13 (23)	9 (33)	4 (14)	>0.001^¶^
	T2aN0M0	15 (27)	12 (44)	3 (10)	
	T2bN0M0	2 (4)	1 (4)	1 (3)	
	T3N0M0	9 (16)	2 (7)	7 (24)	
	T4N0M0	13 (23)	1 (4)	12 (42)	
	T2aN1M0	1 (2)	1 (4)	0	
	T4N1M0	3 (5)	1 (4)	2 (7)	
Tumor size, mm	Median [range]	37 [15-110]	43 [25-110]	30 [15-99]	0.005^§^
	>37 mm	28 (50)	14 (52)	14 (48)	0.789^*^
	≥37 mm	28 (50)	13 (48)	15 (52)	
Tumor number	Single	50 (89)	22 (81)	28 (97)	0.081^¶^
	Multiple	6 (11)	5 (19)	1 (3)	
CEA, mg/ml	Median [range]	4.4 [0.7-51.2]	2.6 [0.7-44.0]	2.3 [1.1-51.2]	0.052^§^
CA19-9, U/ml	Median [range]	124 [0-7614]	190 [0-7614]	94.9 [0-5540]	0.737^§^
Dose/fractionation [BED10]	52.8 Gy (RBE)/12 fr. [76]	1 (2)	0	1 (3)	0.001^¶^
	52.8 Gy (RBE)/4 fr. [122]	5 (9)	5 (19)	0	
	60 Gy (RBE)/12 fr. [90]	7 (13)	6 (22)	1 (3)	
	60 Gy (RBE)/4 fr. [150]	5 (9)	4 (15)	1 (3)	
	64.8 Gy (RBE)/24 fr. [82]	1 (2)	0	1 (3)	
	65 Gy (RBE)/26 fr. [81]	1 (2)	0	1 (3)	
	66 Gy (RBE)/10 fr. [110]	1 (2)	1 (4)	0	
	70.2 Gy (RBE)/26 fr. [89]	13 (23)	4 (15)	9 (31)	
	76 Gy (RBE)/20 fr. [105]	22 (39)	7 (26)	15 (51)	

^*^Chi-square test, ^§^Mann‒Whitney *U* test, ^¶^ Fisherʼs exact test.

Abbreviations: HBV: hepatitis B virus, HCV: hepatitis C virus, BED10, biological effective dose when α/β = 10 is applied, Gy: gray, RBE: relative biological effectiveness, CEA: carcinoembryonic antigen, CA19-9: carbohydrate antigen 19-9.

### Treatment effect

The rate of completion of prescribed CIRT treatment course was 98% (55/56); the one patient with PHC who did not complete the course was required to undergo stent replacement due to worsening cholangitis after delivery of 24 of 25 fractions. The median follow-up time was 10.6 (range 1.6–129.2) months; seven patients (13%) were lost to follow-up. Sixteen patients (28%) experienced local recurrence. The 1- and 2-year LC rates were 79.4% (95% confidence interval [CI], 62.7‒89.2%) and 58.2% (95% CI, 37.7‒74.0%), respectively. Distant failure was noted in 19 patients (34%), including 3 patients who also experienced local failure. The median time to progression for all 56 patients was 9.2 months, with 1- and 2-year PFS rates of 54.1% (95% CI, 38.4‒67.4%) and 32.3% (95% CI, 18.0‒47.6%), respectively. Of the 32 patients who experienced recurrence after CIRT (local failure only, *n =* 13; distant failure only, *n =* 16; local plus distant failure, *n =* 3), 20 (65%) selected best supportive care for treatment. The remaining 12 patients (35%) chose the following salvage treatments: chemotherapy (*n =* 10), radiofrequency ablation (*n =* 1), and repeat CIRT (*n =* 1), respectively.

**Figure 1:**
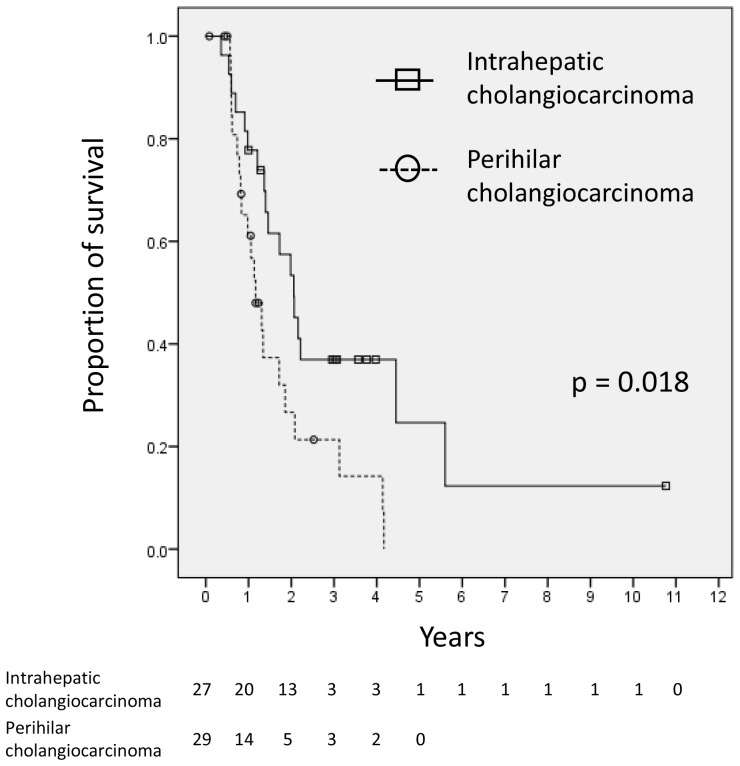
Kaplan‒Meier curves of the OS rates of patients with intrahepatic cholangiocarcinoma (*n* = 27) and perihilar cholangiocarcinoma (*n* = 29). There was a significant difference between the two survival curves (*p* = 0.018).

By the end of follow-up, 15 patients (27%) were still alive. The MST for all 56 patients was 14.8 (range, 2.1-129) months. The 1- and 2-year OS rates were 69.7% (95% CI, 55.3‒80.2%) and 40.9% (95% CI, 26.8‒54.4%), respectively. Figure 1 shows Kaplan‒Meier curves for the OS of the IHC (*n =* 27) and PHC (*n =* 29) patients. The 1- and 2-year OS rates and MST were 77.8% (95% CI, 57.1‒89.3%), 53.4% (95% CI, 32.6‒70.4%), and 23.8 months for the patients with IHC versus 61.1% (95% CI, 39.7‒76.9%), 26.3% (95% CI, 10.1‒45.9%), and 12.6 months for the patients with PHC, respectively (log-rank, p=0.018). After excluding the 7 patients lost to follow-up from the analysis, the 1- and 2-year OS rates and MST of the remaining 49 patients were 67.3% (95% CI, 52.3‒78.6%), and 39.2% (95% CI, 25.4‒52.7%), and 17.5 months, respectively. The MSTs of the patients with IHC (*n =* 26) and PHC (*n =* 23) were 24.7 and 13.6 months, respectively (log-rank, p=0.011). The distributions of the causes of death and local/distant failure in all patients and according to the tumor site (PHC vs. IHC) are presented in Table 3.

Both the univariate and multivariate analyses revealed that cholangitis pre-CIRT and Child‒Pugh class B were significant prognostic factors for an unfavorable OS (Table 2). On the other hand, no prognostic factors among those evaluated were significantly associated with LC or PFS (Supplementary Table 1).

**Table 2:** Prognostic factors for overall survival (OS)

		No. of patients	Univariate analyses		Multivariate analyses				
			1-year OS%	p	HR	95% CI			p
Age, years	>76	33	71.0	0.855					
	>76	23	67.8						
Sex	Male	38	72.1	0.917					
	Female	18	64.7						
Operable	yes	10	60.0	0.411					
	no	46	71.8						
Performance status	0/1	52	69.8	0.389					
	2	4	66.7						
Child‒Pugh class	A	52	73.4	0.014	Reference				
	B	4	25.0		4.079	1.342	‒	12.400	0.013
Prior therapy for cholangiocarcinoma	yes	13	76.9	0.177					
	no	43	67.3						
Cholangitis pre-CIRT	yes	11	60.0	0.012	3.091	1.180	‒	8.100	0.022
	no	45	71.9		Reference				
Biliary stenosis pre-CIRT	yes	27	63.5	0.035	0.416	0.072	‒	2.396	0.326
	no	29	75.1		Reference				
Stent treatment for biliary stenosis pre-CIRT	yes	24	63.0	0.016	3.736	0.745	‒	18.730	0.109
	no	32	78.4		Reference				
Diagnostic method	Imaging	21	68.4	0.871					
	Pathological	35	70.2						
TNM stage	T1-2N0M0	39	75.1	0.133					
	Advanced	17	58.2						
Tumor size	>37 mm	28	65.3	0.933					
	>37 mm	28	74.1						
Tumor number	Single	50	68.5	0.640					
	Multiple	6	80.0						
CA19-9	>200	33	78.1	0.334					
	>200	23	56.5						
CEA	>10	48	71.6	0.868					
	>10	8	57.1						
Total BED10	>105	23	75.9	0.482					
	>105	33	65.6						
Fraction number	>20	20	23.8	0.234					
	>20	36	16.1						

Abbreviations: HR: hazard ratio; CI: confidence interval; CA19-9: carbohydrate antigen 19-9; CEA: carcinoembryonic antigen; BED10: biological effective dose when α/β = 10 is applied.

**Table 3:** Causes of death and local/distant failure

	All patients [n = 56]	Intrahepatic cholangiocarcinoma [n = 27]	Perihilar cholangiocarcinoma [n = 29]
Alive patients, *n* (%)	15 (27%)	8 (30%)	7 (24%)
Patients lost to follow up	7 (13%)	1 (4%)	6 (21%)
Total deaths, *n* (%)	41 (73%)	19 (70%)	22 (76%)
Cholangiocarcinoma-specific death	31	14	17
^#1^ Liver failure due to CIRT (newly developed ascites)	1	1	0
^#2^ Liver failure due to persistent poor liver function	1	1	0
^#3^ *Liver failure due to cholangitis*	1	0	1
^#4^ *Liver failure due to biliary duct stenosis*	1	0	1
*Sepsis due to cholangitis*	3	0	3
Interstitial pneumonia due to chemotherapy	1	1	0
Cholangitis due to gallstones	1	1	0
Senility	1	1	0
Metastasis, n (%)	19 (34%)	10 (37%)	9 (31%)
Liver (only)	9	6	3
Lung (only)	1	1	0
Lymph node (only)	2	1	1
Peritoneum (only)	2	0	2
Liver + lung	1	0	1
Liver + lymph node	1	1	0
Liver + peritoneum	1	0	1
Liver + lung + lymph node	2	1	1
Local recurrence, *n* (%)	16 (29%)	7 (26%)	9 (31%)

Abbreviations: CIRT: carbon-ion radiotherapy

#1‒#4, corresponding to cases #1‒4 listed in Table 4.

The causes listed in italics were observed exclusively among the perihilar cholangiocarcinoma patients.

### Adverse events

Table 4 shows the details of the four patients who died of liver failure. Of these, one patient with IHC (#1 in Table 4) was judged to have CIRT-induced liver disease, as the patient first developed ascites following CIRT, and ascites cytology was negative. He died of liver failure at 4.3 months post-CIRT. Of the remaining three patients, one with IHC (#2 in Table 4) experienced deteriorating liver function due to IHC pre-CIRT and died 14.5 months post-CIRT, and two with PHC (#3 and #4 in Table 4) had persistent cholangitis or biliary stenosis pre-CIRT and died at 7.2 and 22.3 months post-CIRT, respectively. It was difficult to determine the role of CIRT in the liver failure found in cases #2‒4 in Table 4. In addition, there was one grade 3 CIRT-related adverse event of bile duct stenosis, in an IHC case. No other CIRT-related grade 3 or more severe adverse events were observed, including gastrointestinal events such as nausea, vomiting, bleeding, ulcers, or stricture. The relationships between severe adverse events and several factors were analyzed on univariate analysis (Supplementary Table 2), and cholangitis pre-CIRT was the only factor found to be significantly associated with severe adverse events after CIRT (*p* = 0.047).

**Table 4:** Four cases of death caused by liver failure after carbon-ion radiotherapy

#	Age, years	M/F	Pre-CIRT condition	Maximum tumor size, mm	Dose Gy (RBE)	Fr	Tumor site	Symptoms post-CIRT	Time to death, months
1	77	M	TACE performed 2 months pre-CIRT for another lesion	33	76	20	IHC	Newly developed ascites	4.3
2	69	F	ICG R15 of 44%	55	52.8	4	IHC	Continuous gradual deterioration of liver function	14.5
3	76	F	ERBD required for recurring cholangitis	30	70.2	26	PHC	Continuous cholangitis	7.2
4	86	M	ERBD required for biliary duct stenosis and jaundice	15	70.2	26	PHC	Continuous biliary stenosis	22.3

Abbreviations: M: male; F: female; Gy: gray; RBE: relative biological effectiveness; CIRT: carbon-ion radiotherapy; TACE: transcatheter arterial chemoembolization; IHC: intrahepatic cholangiocarcinoma; ICG R15: indocyanine green retention rate at 15 minutes; ERBD: endoscopic retrograde biliary drainage; PHC: perihilar cholangiocarcinoma

## DISCUSSION

The 56 patients with cholangiocarcinoma treated with CIRT, of whom more than 80% were inoperable with comorbidities or advanced tumors as judged by surgeons, demonstrated relatively favorable treatment outcomes, especially for the patients with IHC, without undergoing surgical resection. This study revealed a MST of 14.8 months for all 56 patients, 23.8 months for the 27 patients with IHC, and 12.6 months for the 29 patients with PHC after CIRT for cholangiocarcinoma. To the best of our knowledge, there has been no multicenter study on cholangiocarcinoma post-CIRT.

Among the patients with PHC, two died of liver failure, which was caused by bile duct stenosis (*n =* 1) or cholangitis due to tumor invasion (*n =* 1), and three died of sepsis caused by cholangitis (these three causes are indicated in italics in Table 3). Liver failure or sepsis following bile duct stenosis or cholangitis can occur during the natural course of PHC [2], which can confound evaluation of the treatment effects in PHC after CIRT. Nonetheless, the OS was significantly poorer among patients with PHC compared with those with IHC in the present study. Bile duct stenosis and cholangitis pre-CIRT were observed in the patients with PHC, and remain even after CIRT, which may affect prognosis directly. Such features of PHC may explain why cholangitis pre-CIRT was found to be significant factor for OS in this study.

Furthermore, OS was significantly poorer among patients with Child‒Pugh class B compared with class A, although this result was based on only four patients with Child‒Pugh class B in this cohort; however, a relationship between Child‒Pugh class B and poor survival is supported by a study on CIRT for hepatocellular carcinoma [14]. Close monitoring of patients with Child‒Pugh class B cholangiocarcinoma undergoing CIRT will be needed.

Table 5 presents a comparison of our results with those from other trials using photon or proton radiotherapy administered with or without chemotherapy. The OS rates and MST of the present study are comparable with those in previous reports on proton therapy or SBRT; CIRT may also be a promising therapy for patients with cholangiocarcinoma who cannot undergo surgery.

**Table 5:** Comparison of survival outcomes between the present study, and previous studies of radiotherapy

Treatment, Author	Year	*n*	Median total dose [Gy or Gy(RBE)]	Median fraction	Median BED10	Overall survival (%)		MST (mo)
						1-year	2-year	
**Intrahepatic cholangiocarcinoma^a^**								
Photon radiotherapy								
SBRT/IMRT								
Tse *et al*. [21]	2008	10	36	6	58	58^e^	NA	15^e^
Ibarra *et al*. [22]	2012	11	30	3	60	45^f^	NA	11^f^
Tao *et al*. [23]	2016	79^b^	58	15	80	87^g^	61^g^	30^g^
Shen *et al*. [24]	2017	28	45	3–5	>100	57^f^	32^f^	15^f^
Conventional RT								
Zeng *et al*. [25]	2006	38	50	25	60	50.1^g^	11.8^g^	NA
		37	Non-EBRT	-	-	24.8^g^	5.5^g^	NA
Chen *et al*. [26]	2010	35	50	25	60	38.5^g^	9.6^g^	9.5^g^
		49	Non-EBRT	-	-	16.4^g^	4.9^g^	5.1^g^
Kim *et al*. [27]	2013	25	44.7 (CCRT)	2‒3Gy/fr.	54^d^	30.4^g^	NA	9.3^g^
		67	Chemotherapy	-	-	22.4^g^	NA	6.2^g^
Proton radiotherapy								
Ohkawa *et al*. [28]	2015	12^c^	72.6	22	97	82^f^	61^f^	27.5^f^
Hong *et al*. [29]	2016	39	58	15	80	69.7^f^	46.5^f^	22.5^f^
Carbon-ion radiotherapy								
(present study)	2019	27	76	20	105	77.8^f^	53.4^f^	23.8^f^
**Perihilar cholangiocarcinoma**								
Photon radiotherapy								
SBRT								
Momm *et al*. [7]	2010	13	48	4	106	NA	NA	23.6^g^
Kopek *et al*. [16]	2010	27^h^	45	3	112	NA	NA	10.6^f^
Polistina *et al*. [8]	2011	10	30	3	60	NA	80^g^	35.5^g^
Conventional X-ray RT ± brachytherapy ± chemotherapy								
Ghafoori *et al*. [30]	2011	37^i^	45	1.8-3Gy/fr	NA	59^f^	22^f^	14 ^f^
Chen *et al*. [6]	2015	16	54 (CCRT)	1.8-2 Gy/fr	65^d^	41^f^	NA	13.5^f^
		18	54 (RT alone)				NA	6.7^f^
Carbon-ion radiotherapy								
(present study)	2019	29	76	20	105	63.5^f^	27.3^f^	12.6^f^

^a^According to differences in the definition of intrahepatic cholangiocarcinoma, some cases of perihilar cholangiocarcinoma may be included among these studies.

^b^Various radiotherapy modalities such as proton radiotherapy, IMRT, conventional radiotherapy were included.

^c^Curative treatment number

^d^Calculated according to 2 Gy/fraction

^e^The starting day was not available for measuring survival time.

^f^Survival time was measured from the start of radiotherapy

^g^Survival time was measured from the time of diagnosis.

^h^One case of intrahepatic cholangiocarcinoma was included in this study.

^i^Six patients were performed brachytherapy only.

Abbreviations: Gy: gray; RBE: relative biological effectiveness; BED: biological effective dose; MST: median survival time; NA: not available; RT: radiotherapy; SBRT: stereotactic body radiotherapy; IMRT: intensity modulated radiotherapy; EBRT: external beam radiotherapy; CCRT: concurrent chemoradiotherapy; fr.: fraction.

Nevertheless, the treatment outcomes were inadequate in our study, as the distant and local failure rates were 34% and 29%, respectively (Table 3), likely contributing to the poor survival. Regarding the CTV margin, Bi et al. reported pathological microinvasions 0.4-8 mm from the gross tumor in IHC cases [15], suggesting that a CTV margin of 10 mm is sufficient in most cases. However, the actual CTV margin in some cases of this study was less (median 4 mm; range 0–10 mm) to avoid adjacent organs at risk, which may have affected the treatment outcomes reported here. In the present study, only one patient received concurrent chemotherapy. By comparison, in a conventional radiotherapy series, OS was significantly improved when treatment included concurrent chemotherapy compared with RT alone [6]. These results suggest that CIRT combined with concurrent chemotherapy may be a reasonable treatment strategy for improving outcomes in patients with unresectable cholangiocarcinoma. However, since the safety of the combined treatment is unknown, a prospective study is required.

Concerning adverse events, four patients died of liver failure (Table 4). One of these was judged to be CIRT-related liver failure due to newly developed ascites without disease recurrence post-CIRT, although transcatheter arterial chemoembolization was performed for another lesion 2 months before CIRT and may have played a role. The remaining three liver failure cases had low liver function with a high indocyanine green retention rate at 15 minutes or biliary symptoms such as cholangitis or bile duct stenosis, due to progression of their tumor prior to CIRT. Therefore, it is difficult to determine whether three liver failure cases are related to CIRT. In addition, bile duct stricture was observed in one patient with IHC as a CIRT-related severe adverse event. However, no gastrointestinal severe adverse events were noted in this study, which may be due to the highly conformal nature of the dose distribution of CIRT compared with photon radiotherapy, including SBRT [8, 16]. Therefore, the overall safety of CIRT is generally acceptable. Caution should be used in patients who experience cholangitis pre-CIRT, considering the potential for severe adverse events identified in this study.

There were limitations to this study. First, this study was retrospectively conducted in a limited number of patients. Second, several dose and fractionation regimens were included, with a large BED range from 76 (52.8 Gy [RBE]/12 fractions) to 150 (60 Gy [RBE]/4 fractions). Standardization of the treatment regimen will be needed for efficacy evaluation in large-scale future studies. Third, there was a relatively high rate of lost to follow-up cases (13%), and salvage therapy was performed in 12 patients who experienced recurrence, which might have affected the results of this study. Fourth, this study did not include cases of distal extrahepatic cholangiocarcinoma of the bile duct; therefore, the safety and efficacy of CIRT for distal ductal disease are still unknown. Fifth, the median follow-up period was too short to evaluate the long-term safety and efficacy of CIRT. Despite the limitations, this multicenter study is the largest analysis of CIRT for cholangiocarcinoma to date.

In conclusion, this multicenter study in Japan showed promising efficacy and safety for CIRT in patients with cholangiocarcinoma who had not undergone surgery including inoperable cases.

## MATERIALS AND METHODS

### Study design

In December 2018, the data of patients with cholangiocarcinoma who were treated with CIRT between 2005 and 2016 were analyzed retrospectively in J-CROS 1703 (UMIN 000035565). Overall survival (OS) was the primary endpoint, whereas local control (LC), time to progression, progression-free survival (PFS), completion rate of the prescribed CIRT treatment course, and evaluation of adverse events were the secondary endpoints.

Seventy-five patients with cholangiocarcinoma were treated with CIRT at Hyogo Ion Medical Center (Tatsuno, Japan), Hospital of the National Institute of Radiological Sciences (Chiba, Japan), Gunma University Heavy Ion Medical Center (Maebashi Japan), and SAGA HIMAT Foundation Ion Beam Therapy Center (Tosu, Japan) between 2005 and 2016. This multicenter retrospective study was approved by institutional review board of each participating institution. The protocol was performed in accordance with the Declaration of Helsinki [17].

### Eligibility

The inclusion criteria for entry into this study were as follows: 1) biopsy-proven cholangiocarcinoma, or a definitive diagnosis by dynamic contrast-enhanced computed tomography/magnetic resonance imaging studies together with elevated tumor markers (CA19-9 and/or CEA), 2) all lesions, including the primary tumor, daughter nodule, and lymph node metastasis, were treated in a single radiation field, 3) a measurable lesion, 4) no metastasis to other organs, 5) absence of active double cancers other than cholangiocarcinoma, 6) a hepatic disorder classified as Child‒Pugh class A or B, 7) age >20 years, 8) a performance status of 0 to 2 on the Eastern Cooperative Oncology Group scale, and 9) ability to understand and sign an informed consent form at each institution.

The exclusion criteria for this study were as follows: 1) previous treatment of the target tumor by other radiation therapies or surgery, 2) tumor invasion of the digestive tract, 3) untreatable ascites, 4) active malignant tumors other than the cholangiocarcinoma to be treated, 5) severe comorbidities such as uncontrolled diabetes mellitus, renal failure, or cardiac failure, and 6) a serious medical or psychological condition precluding safe administration of treatment.

Of the 75 patients treated with CIRT at the four institutions, 19 did not meet the eligibility criteria for this study for the following reasons: previous surgical treatment of the cholangiocarcinoma (*n =* 7), diagnosis by imaging without elevated tumor marker (CA19-9 or CEA) levels (*n =* 5), presence of metastasis to other organs (*n =* 3), presence of another active cancer (*n =* 2), Child‒Pugh class C (*n =* 1), and no measurable lesion (*n =* 1). The remaining 56 patients were included in this study.

### Treatment

The CIRT doses were calculated by multiplying the absorbed dose of the carbon ions by the RBE. The RBE value of carbon ions was assumed to be 3 at the distal part of the spread-out Bragg peak. Carbon-ion beams were controlled using with a pair of wobbler magnets, beam scatterers, ridge filters, a range shifter, multi-leaf collimators, and a range compensator [11].

The margin of the clinical target volume was established as 10mm from the tumor. The planning target volume was determined by adding an additional margin of 5–10mm. If the tumors was too close to organs such as the gastrointestinal (GI) tract, these margins were reduced to avoid over-irradiation of organs at risk. The exact margin was decided on a case-by-case basis by the treating physician.

Dose prescriptions were selected according to the proximity of the tumor to the GI tract. At three of the four institutions, 52.8 Gy (RBE) and 60.0 Gy (RBE) were routinely administered via 4-fraction CIRT. If the tumor is close to the GI tract, 12-fraction CIRT is used. At the fourth institutions, a dose of 76 Gy (RBE)/20 fractions or 60 Gy (RBE)/10 fractions is prescribed in normal cases, while a dose of 70.2 Gy (RBE)/26 fractions, 65 Gy (RBE)/26 fractions, or 64.8 Gy (RBE)/25 fractions were prescribed in cases in which the tumor was close to the GI tract. On the other hand, neither tumor size nor liver function was considered in determining the dose prescription.

Patient‒machine alignment was achieved by overlapping the onboard image, taken in a daily vertical/horizontal position using a kV X-ray, with the reconstructed two-dimentional image taken during planning CT. Deviations in the skeletal anatomy, diaphragm, and implantation of fiducial markers were minimized between the two images. Respiratory gating at the end of the exhalation phase was used for CT planning, verification of the position on the treatment board, and irradiation [18]. Written informed consent was obtained from all enrolled patients.

### Evaluation

PHC is defined as cholangiocarcinoma located in any of the right, left or common hepatic duct. IHC is defined as cholangiocarcinoma located peripheral to the secondary bifurcation of the right and/or left hepatic duct. LC was defined as no evidence of tumor regrowth within the planning target volume. The LC time was defined as the interval between the start of CIRT and local failure diagnosis or the last follow-up. Progression free was defined as neither evidence of tumor regrowth nor other recurrence. Survival time was calculated as the interval between the start of CIRT and death or the last follow-up. Death from hepatic failure related to CIRT was defined as death caused by deterioration of liver function without progression of the cholangiocarcinoma and without symptoms such as cholangitis or biliary stricture. In normal tissues, adverse events were classified according to the National Cancer Institute Common Terminology Criteria for Adverse Events, version 4.0 [19].

### Statistical analyses

The OS, LC, and PFS rates were evaluated using the Kaplan‒Meier method. For univariate analyses, log-rank tests were used to compare OS, LC, and PFS between subgroups. All factors with a *p*-value <0.1 in the univariate analysis were included in a multivariate analysis using the Cox proportional hazards model. Tumor site (IHC or PHC) was excluded from the prognostic factor analysis because these two types of cholangiocarcinoma are inherently different. Proportions were compared using the chi-square test or Fisher’s exact test. The equality of population medians between cohorts was evaluated using the Mann‒Whitney U test. The optimal cutoff values for the BED10, based on the maximum Youden index [20], for predicting local failure and severe adverse events were determined to be 105 and 89 Gy, respectively, by receiver operating characteristics analysis. A *p*-value <0.05 was considered to represent significance. All statistical analyses were performed using SPSS software (version 20.0; IBM Japan, Ltd, Tokyo, Japan).

## SUPPLEMENTARY MATERIALS



## Abbreviations

BED: biological effective dose
CIRT: carbon-ion radiotherapy
IHC: intrahepatic cholangiocarcinoma
GI: gastrointesitinal
J-CROS: Japan Carbon-Ion Radiation Oncology Study Group
LC: local control
MST: median survival time
OS: overall survival
PFS: progression-free survival
PHC: perihilar cholangiocarcinoma
RBE: relative biological effectiveness
SBRT: stereotactic radiotherapy
