# Supplementary material for: Carbon-ion radiotherapy for cholangiocarcinoma: a multi-institutional study by and the Japan carbon-ion radiation oncology study group (J-CROS)
Source: Oncotarget. 2019 Jul 9;10(43):4369–79. doi: 10.18632/oncotarget.27028 (PMC6633891; doi:10.18632/oncotarget.27028)
Supplement: Supplementary file 1 [file oncotarget-10-4369-s001.pdf]

Carbon-ion radiotherapy for cholangiocarcinoma: a multi-institutional study by the Japan carbon-ion radiation oncology study group (J-CROS)

SUPPLEMENTARY MATERIALS

Supplementary Table 1: Prognostic factors for local control and progression-free survival

			Local control		Progression free survival	
			Univariate analyses		Univariate analyses	
		No. of patients	1-year (%)	*p*	1-year (%)	*p*
Age, years	<76	33	74.5	0.855	42.2	0.150
	≥76	23	87.1		72.7	
Sex	Male	38	78.2	0.214	58.0	0.905
	Female	18	82.5		46.2	
Operability	yes	10	100	0.294	78.7	0.411
	no	46	74.8		31.7	
Performance status	0/1	52	78.1	0.925	53.9	0.853
	2	4	100		35.4	
Child–Pugh class	A	52	81.1	0.347	56.3	0.567
	B	4	0		0	
Prior therapy for cholangiocarcinoma	yes	13	68.3	0.354	42.7	0.348
	no	43	83.4		58.2	
Cholangitis pre-CIRT	yes	11	44.4	0.080	36.4	0.384
	no	45	87.3		58.4	
Biliary stenosis pre-CIRT	yes	27	69.3	0.289	53.0	0.511
	no	29	86.1		54.5	
Stent treatment for biliary stenosis pre-CIRT	yes	24	70.0	0.365	50.7	0.285
	no	32	83.6		55.7	
Diagnostic method	Imaging	21	71.8	0.884	37.6	0.123
	Pathological	35	83.7		64.1	
TNM stage	T1-2N0M0	39	82.4	0.650	60.8	0.252
	Advanced	17	71.3		35.8	
Tumor size	<37 mm	28	78.2	0.781	49.7	0.670
	≥37 mm	28	80.9		58.5	
Tumor number	Single	50	76.9	0.568	55.7	0.471
	Multiple	6	100		40.0	
CA19-9	<200	33	83.5	0.915	53.1	0.345
	≥200	23	72.7		54.9	
CEA	<10	48	78.8	0.454	56.2	0.888
	≥10	8	83.3		42.9	
Total BED	<105	23	81.6	0.765	51.8	0.846
	≥105	33	77.8		55.7	
Fraction number	<20	20	81.8	0.857	58.2	0.662
	≥20	36	77.2		51.8	

HR: hazard ratio; CI: confidence interval; CA19-9: carbohydrate antigen 19-9; CEA: carcinoembryonic antigen; BED: biological effective dose.

Supplementary Table 2: Factors predicting severe adverse events

Factor		*P*[*]
Age, years	<76 vs ≥76	0.085
Sex	Male vs female	0.479
Operability	Yes vs no	0.093
Performance status	0/1 vs 2	0.606
Child–Pugh class	A vs B	0.680
Prior therapy for cholangiocarcinoma	Yes vs no	0.672
Cholangitis pre-CIRT	Yes vs no	0.047
Biliary stenosis pre-CIRT	Yes vs no	0.154
Stenting therapy for biliary stenosis pre-CIRT	Yes vs no	0.363
Diagnostic method	Imaging vs pathological	0.267
TNM stage	T1-2N0M0 vs advanced	0.603
Tumor size, mm	<37 vs ≥37	0.500
Tumor number	Single vs multiple	0.445
CA19-9, U/ml	<200 vs ≥200	0.309
CEA, mg/ml	<10 vs ≥10	0.552
Total BED	<89 vs ≥89	0.670
Fraction number	<20 vs ≥20	0.099

[*]Fisher's exact test

HR: hazard ratio; CI: confidence interval; CA19-9: carbohydrate antigen 19-9; CEA: carcinoembryonic antigen; BED: biological effective dose.
